# The Two Narcotic Laws

**Published:** 1917-09

**Authors:** 


**A Monthly Review of Medicine and Surgery**

EDITOR AND PUBLISHER, DR. A. L. BENEDICT, 228 Summer St., corner of Elmwood Ave., Buffalo, (Address for all communications. Please make personal and telephone calls before 1 P. M.)

Third, in age, on the western continent. Independent, but supports professional organizations. News limited mainly to Western N. Y. and Penn.

Subscription $2.00 a year; $3.00 for two years in advance. Changes and errors in address or failure or duplication of delivery, should be reported immediately.

Yearly Volume 73 SEPTEMBER, 1917 Number 2

### The Two Narcotic Laws.

There are laws against arson, adequate and fairly well inforced. On the whole, we are inclined to think that fires are as few and incendiaries as frequently apprehended as if we had a law that required a license for the sale or possession of matches, kerosene, cans that might presumably be used to hold kerosene, etc., and that made it necessary for every one using such articles, however, innocently, pay a tax, have an annual license, and to purchase, sell or give away such articles, through the medium of special blanks. Moreover, we incline strongly to the belief that there would be no diminution in fires if a board who had never seen a fire, were impowered to rule that vaseline was kerosene, that cold cream was not, and that crinoline was subject to the regulations as to purchase, sale and use, because the word sounded like kerosene. By analogy, we would expect such a board to rule that a licensee who arranged kindling and coal in a stove and touched the kindling with a match, started a fire personally when he did it in somebody else's house but did not personally do so when he performed the same feat at home.

However, opinions differ in regard to the best means of attaining an end and, we may granting that the best way of eliminating indulgence in narcotics is not to get after the addict nor to use the honest physician as a trusted assistant of the law, but to tag the individual pill and spoonful and tax the physician, druggist and every one else concerned. With this assumption, both the nation and the state have satisfactory laws on the subject—with certain minor qualifications. For instance, as our country is at present organized, the nation had no right to legislate at all with regard to local practice but it got around this little constitutional provision by imposing a tax. There was no good reason or precedent for imposing a tax of this sort, any more than for imposing a tax on any other legitimate business, under such circumstances that practically every one in the business would be compelled to pay it and that no considerable benefit could be derived in the way of revenue. A quite incidental objection followed, that the law like most laws which do not apply to obvious crimes, but which are artefacts of a complicated civilization, had to be interpreted. The interpretation of the law was left to an office force, unquestionably well disposed, conscientious and generally well informed but not technically trained in pharmacology. Inevitably, some of the interpretations, as in that apomorphine was a habit-producing narcotic and that the direct administration of a drug by a physician to a patient was personal attendance at the residence of the patient and not personal attendance at the physician's office, were at variance with ordinary professional judgment. One is reminded of the ruling of an English guard with regard to the status of pets carried on trains: "Cats are dogs; parrots are chickens; turtles are insects and travel free."

Very gradually, the law is being interpreted in conformity with facts and it is not being inforced or punishment meted out, on purely technical points. Backed by a really efficient detective effort to get at actual violations of the spirit of the law, in other words, by carrying the general principles of compulsory reform as against crime and malpractice in general, the law is being inforced as well as if it had not been enacted in its present form at all. In addition, the checking and counterchecking of narcotics certainly has rendered them much less easy of access—to such a degree in fact that many have committed suicide and a good many addicts and those suffering temporary pain, have suffered for lack of narcotics.

It may seem like pure contrariness to object to the national interference in local matters and then to object to the state's virtually duplicating the national narcotic law, and further to object because the duplication is not exact. Practically, however, it would have simplified matters if the state had either omitted or repealed all legislation on the matter, or had merely endorsed the action of the national government or, if a state law had to be passed, to have made the duplication exact.

Some physicians have gone so far as to declare that they would cut the Gordian knot by dropping narcotics altogether and letting patients suffer. But even so radical a course is impracticable, quite aside from the occasional necessity of using narcotics. Without a license, the physician has not even the privilege of turning his stock of narcotics—including some rather harmless samples—over to a hospital or of destroying them. He will be surprised to find that some proprietary drug that he uses or that is pressed upon him by an agent, contains a narcotic, or that some ruling classifies as a narcotic some synthetic drug which he does not conceive to be so. At any rate, it is cheaper to pay the two dollar taxes than to be investigated as a suspect. July 1 is the time for registering, paying the fees and filing an inventory. A reasonable, innocent delay will probably be overlooked. A copy of the inventory, the licenses themselves and copies or memoranda of all prescriptions or administrations of any drug that can possibly be considered a narcotic, should be kept. Purchases of drugs require special blanks but any misunderstanding of the law as regards prescriptions for patients or for purchase for dispensing, will probably be corrected by druggists. However, the laws should be diligently studied and saved for reference.

